# Individual and Store Characteristics Associated with Brand Choices in Select Food Category Redemptions among WIC Participants in Virginia

**DOI:** 10.3390/ijerph14040364

**Published:** 2017-03-31

**Authors:** Qi Zhang, Chuanyi Tang, Patrick W. McLaughlin, Leigh Diggs

**Affiliations:** 1 School of Community and Environmental Health, Old Dominion University, Norfolk, VA 23529, USA; leighanndiggs@gmail.com; 2 Department of Marketing, Old Dominion University, Norfolk, VA 23529, USA; ctang@odu.edu; 3 United States Department of Agriculture—Economic Research Service, Washington, DC 20024, USA; patrick.mclaughlin@ers.usda.gov

**Keywords:** Women, Infants, and Children (WIC), brand choices, infant fruits and vegetables

## Abstract

The Special Supplemental Nutrition Program for Women, Infants, and Children (WIC) often allows participants to redeem food benefits for various brands at different costs. To aid the program’s food cost containment efforts, it is important to understand the individual and store characteristics associated with brand choices. This study used the WIC Electronic Benefit Transfer (EBT) data for 239,062 Virginia WIC participants’ brand choices in infant fruits and vegetables (F&Vs) and whole grain bread in May 2014–February 2015, one of the first such data sets available in the U.S. for research purposes. Mixed effects logistic regression models were used to analyze the choice of higher-priced brands over lower-priced brands. Minority participants were significantly more likely to redeem higher-priced brands of infant F&Vs, but more likely to choose lower-priced brands of bread. Participants shopping in urban stores or midsized stores (with 5–9 registers) were less likely to choose higher-priced brands compared to rural stores or large stores (with 9+ registers). Race/ethnicity and store characteristics may be significant factors in participants’ brand choices. The results can help develop interventions that encourage targeted participants to redeem lower-priced but equivalently healthy brands. This may not only help contain WIC program costs, but help participants manage their own non-WIC food expenses as well.

## 1. Introduction

The Special Supplemental Nutrition Program for Women, Infants, and Children (WIC) is one of the largest food assistance programs in the U.S. [1]. A key focus of the WIC program is to provide nutritionally targeted foods, or food packages, at no cost to low-income pregnant and post-partum women, infants up to one year of age, and children up to five years of age, in addition to nutrition education and health interventions, especially for the support of breastfeeding. Different classes of WIC participants are prescribed different food packages providing different quantities of foodstuffs, rather than being awarded a dollar amount [2]. The WIC program serves approximately 8 million Americans monthly, including 50% of newborn infants and 25% of children nationwide [3]. The positive health impact of WIC participation is well documented [4,5].

The WIC program is administrated at both the federal and the state level. The U.S. Department of Agriculture (USDA), the federal WIC administrative agency, mandates nutritional criteria for the program (e.g., only whole grain bread is allowed) and provides grants to the states, who manage their own WIC programs. The national cost of the food package benefits in WIC alone was close to $4.6 billion in 2016 [1]. Federal regulations require state WIC agencies to manage a food benefit distribution system, which includes determining individuals’ WIC eligibility, prescribing food benefits to participants, and authorizing private food vendors with whom participants can redeem their WIC food benefits.

Because WIC is not an entitlement program, the U.S. Congress does not set aside funds for every eligible individual to participate. Instead, states must contain the food and administrative costs within the Congress-authorized budgets each year, i.e., “cost containment” within the WIC program. Food cost accounts for approximately 70% of the total WIC program budget [6]. States must estimate funding for foods in packages that are consistent with the Dietary Guidelines for Americans, readily available, and commonly consumed. The state agents need to also take into account cultural preferences and provide incentives for families to encourage them to participate in the WIC program [7]. Cost containment is an ongoing policy issue, since balancing costs with healthy food choices poses a challenge to the WIC program [3,8]. The need for cost containment stems from the fact that participants can redeem any WIC-eligible food item regardless of the listed price, and hence participants have no explicit economic incentive to be cost conscious. In fact, one study found that when participants purchased ready-to-eat breakfast cereal with their WIC benefits, the cost was 26.3% higher on average than when purchasing with their own money [9]. In an effort to limit food costs, nearly all state agencies exclude some food brands that are deemed “too costly” yet meet federal nutritional guidelines. The degree of brand restriction varies widely across states and product categories. For instance, Virginia WIC allows whole wheat bread brands with a wide price range, while Texas WIC requires the vendors to maintain 85% of their WIC redemptions from the Least Expensive Brands for selected food products, such as milk or cheese [10].

However, restricting participants’ food brand options may have unintended negative consequences for the program, such as discouraging potential enrollees from joining if they perceive they cannot redeem desirable food brands. This concern is especially pressing as participation levels are the lowest since the Great Recession of 2008–2009 [3]. Policy makers remaining under pressure to contain food costs are looking into the subtle influences of brand restriction on participants’ brand choices. Insights from behavioral economics suggest the possible strategy of “nudging” participants toward less costly brands [11]. For example, placing the lower-cost food item brands on more prominent shelf positions may promote WIC participants to redeem them over the higher-cost brand alternatives. These types of behavioral economics-based interventions have been successfully implemented to promote healthy food consumption without limiting consumers’ food choices [12,13]. Thus, considerable interest has arisen in developing behavioral economics interventions for the WIC program to aid in cost containment [14]. However, little is known about the basic factors that influence participants’ brand choices, such as individual characteristics or the types of vendors they patronize while redeeming their benefits [15]. 

The primary challenge to understanding WIC participants’ brand choices, even in basic ways, is the lack of WIC redemption data with Universal Product Codes (UPCs), often referred to as the bar code on the product package. This study took advantage of the WIC Electronic Benefit Transfer (EBT) data in Virginia, one of the first available sets of state-level WIC administrative data in the U.S., which has individual-level redemption records with detailed UPC codes to determine the exact brands redeemed. After reviewing the 17 food categories eligible in Virginia’s WIC program, we selected infant fruits and vegetables (“infant F&Vs” hereafter) and whole wheat bread (“bread” hereafter). These two product categories jointly account for $7,873,342 in redemptions over the study period, with considerable variation in the costs of eligible brands. Infant F&Vs and bread are ideal for this study’s purposes because Virginia WIC allows participants to choose among multiple brands with different costs as opposed to a single designated brand.

The purpose of this study is to examine what participant- and vendor-specific factors influence whether or not a participant chooses higher-cost brands rather than lower-cost alternatives. To the best of our knowledge, this is one of the first studies in the U.S. using transaction-level state WIC EBT data, and it therefore provides a rare opportunity to study WIC participant’s redemption behaviors. The NASEM recommends the use of EBT data in their latest guidelines [7]. As more transaction-level state WIC EBT data become available due to the congressional mandate in the Healthy, Hunger-Free Kids Act of 2010 that requires all WIC state agencies to implement the EBT delivery method by 2020, this study may be used as a preliminary resource to identify cost-saving intervention targets.

## 2. Materials and Methods

### 2.1. Data Sources

The Virginia Department of Health (VDH) provided WIC EBT transaction records spanning from when the VDH fully implemented the EBT system in May 2014 to February 2015. These data contain highly detailed information on WIC EBT transactions involving approximately 130,000 participant households shopping at around 850 authorized WIC vendors. In particular, the data include the Universal Product Code (UPC), quantity, and price of the WIC items purchased; the authorized vendor completing the transaction and the date it took place; and selected characteristics of participants and authorized vendors, including number of participants in a household, race/ethnicity, primary language spoken at home (English, Spanish, and/or other), number of months participating in the WIC program (≤3, >3−≤6, >6–≤9, ≥9 months), vendor locations in urban or non-urban areas, and vendor sizes. The urban/non-urban designation was based on the Isserman model, which integrates the population from the 2000 census tract and the rural/urban county continuum codes from the U.S. Office of Management and Budget (OMB) [16]. The vendor size is defined based on the number of cash registers, as larger vendors may tend to employ more registers to accommodate higher volumes of sales; this is a modeling choice used in previous work [17]. We classified vendors into small, medium, and large vendors depending on whether they have one to 4, 5–9, or >10 registers, respectively. 

### 2.2. Statistical Analyses

The focus of the analysis is on the redemption of 4-ounce containers of infant F&Vs and 16-ounce packages of whole wheat bread. To ensure the studied brands were widely available to participants, we excluded the brands that accounted for <1% of the share of statewide redemptions in each product category. This narrowed our analysis to the redemption of 2 brands in infant F&Vs and 10 brands of whole wheat bread (Table 1). For confidentiality purposes, we are not able to disclose specific brand names. Instead, we used numbers (e.g., Brand 1 and Brand 2) for infant fruits and vegetables’ brands and alphabets (e.g., Brand A and Brand B) for bread brands.

For each food product category, we identified the costliest prominent brand, that is, the brand with the highest average unit cost and a significantly large share of WIC redemptions. For infant F&Vs, the costliest prominent brand is Brand 1, which is the most widely chosen by participants and bears a price that is on average 11.29% more than the other eligible brand, Brand 2. For whole wheat bread, Brand F is the costliest, but it comprises a minuscule 1.44% of the redemption share in this category. Brand A, on the other hand (38.34%), is the most frequently redeemed bread brand and has the second-highest unit cost of redemption. Therefore, we treat Brand A as the costliest prominent brand of bread. From the behavioral economics perspective, participants made a choice given the restricted environment, i.e., there is a framing effect. Therefore, we used the brand choice as the outcome variable. Moreover, we dichotomized the brands into Brand 1 vs. Brand 2 for infant F&Vs and Brand A vs. other brands for bread, since these two types of brands were available at all vendors.

Since households redeem WIC benefits on a monthly basis, we employed a mixed effects logistic regression model with a random effect for households and used participant and vendor characteristics to predict WIC participants’ choice of the most expensive prominent brands. Because redemption behaviors may vary in different vendor-specific store environments, we ran separate regression models for each of the top three retail chains accounting for the highest number of Virginia WIC redemptions. Due to confidentiality requirements, we are unable to disclose the identity of the food chains. We instead refer to each store chain in order of total Virginia WIC market share: Alpha, Beta, and Sigma. These vendors are supermarket chains with a customer base comprising lower- and middle-income consumers. We estimated the mixed effects logistic regression models with clustered standard errors. We used Stata 12 in the statistical analyses (StataCorp, College Station, TX, USA) and subsequently reported odds ratios (ORs), 95% Confidence Intervals (95% CI), and *p*-values.

## 3. Results

### 3.1. Descriptive Statistics of Brands in Infant F&Vs and Whole Grain Bread

The total sample includes 239,062 Virginia WIC participants in 113,341 households who shopped at 856 WIC-approved vendors in May 2014–February 2015. Besides identifying whether or not a given brand is a store brand (e.g., private label), Table 1 summarizes the total value of redemptions of infant F&Vs and bread by brands. In general, participants purchased more of the costliest prominent brand than the other brands for both product categories. Among the infant F&Vs brands, Brand 1 accounted for 70.67% of the roughly $2,193,575 worth of items and 68.46% of the redeemed units. For whole wheat bread, the total redemption cost of the top 10 bread brands was $2,388,434. Most of these brands are national brands. Participants tended to choose national brands over eligible store brands for bread. The average price of the store brands is 59.72% of the cost of national brands. There was a 20% difference in the unit cost between the two most widely chosen bread brands (Brand A vs. Brand B).

### 3.2. Redemption Patterns among Virginia WIC Participants

Table 2 presents the descriptive statistics of the characteristics and redemption patterns of the participants who redeemed infant F&Vs and bread. For infant F&Vs, a total of 40,285 households redeemed more than 3 million units with a total cost of $2,109,324. We observed significant variation in the prevalence of the higher-price Brand 1 redemptions across demographic and household characteristics. Among these households, the two- or three-participant households were the most predominant recipients of infant F&Vs, redeeming 56.5% and 29.0% of all units, respectively. The larger a household was, the more likely they were to choose Brand 1 over Brand 2. A higher proportion of non-Hispanic black (67.2%) and Hispanic (68.2%) households chose Brand 1 compared to non-Hispanic white households (56.6%). Roughly 70% of Spanish-speaking households chose Brand 1, compared to 61.6% of English-speaking households. Brand 1 redemptions increased as participants stayed longer in the WIC program.

For bread, 99,980 WIC households redeemed 1,622,533 units at a total cost of $5,764,018. Much like infant F&Vs, the frequency of the redemption of the costliest prominent brand of bread co-varied with participant characteristics. The percent of households redeeming the higher-price Brand A increased as the number of participants in the households increased. Non-Hispanic whites chose Brand A bread more often than did other ethnicities. English-speaking households also chose Brand A more often than Spanish-speaking households. The tendency to choose Brand A increased in participants’ first three months in the program and then decreased as the participant stayed longer in the program. For example, 41.2% of the households that stayed in the program for 3–6 months chose Brand A, while only 38.4% of households that stayed in the program for more than 9 months did so.

### 3.3. Participant and Store Predictors of Brand Choices for Infant F&Vs

Table 3 presents the estimated mixed effects logistic regression models of the choice of the costliest prominent brand of infant F&Vs (Brand 1). In the pooled (all vendors) model, participant characteristics such as race/ethnicity and the primary language spoken at home have statistically significant relationships with the choice of the higher-price Brand 1. For example, non-Hispanic black and Hispanic households were, respectively, 14% and 27% on average more likely to select this brand compared to non-Hispanic white households. Participants who primarily speak Spanish at home were on average 21% more likely to choose Brand 1 than those speaking English. In addition, the propensity to choose Brand 1 systematically varies with observed vendor characteristics. Participants were on average 37% more likely to choose Brand 1 at small vendors than at large ones, but 9% less likely to choose Brand 1 at vendors located in urban areas than at those in rural locations.

The relationship between participant characteristics and the choice of the higher-cost brand in infant F&Vs varied among the top three retail chains, indicating that unobservable vendor characteristics may be important factors as well. For instance, the relationship between the length of participation in Virginia WIC and infant F&V brand choice was statistically significant in Vendor Beta and Vendor Sigma. In Vendor Sigma, participating in Virginia WIC for longer than three months corresponded to a greater likelihood to redeem Brand 1, but this relationship was not significant in Alpha or Beta. Households made up of four or more participants were 16% less likely to choose Brand 1 compared to single-participant households in Vendor Sigma, but were 7% more likely to do so in Beta outlets.

Furthermore, the strength of the relationships between participants’ race/ethnicity and brand choice varied among retail chains. For example, while minority participants had a higher likelihood of choosing Brand 1 at each retail chain, the magnitude of each coefficient varied. Non-Hispanic black participant households were on average 18% more likely to choose Brand 1 than non-Hispanic white peers when shopping at Vendor Sigma, but 49% more likely to do so at Vendor Beta. A similar pattern occurred at those vendors when comparing Spanish- versus English-speaking households.

Table 4 presents the mixed effects logistic regression estimates for whole wheat bread. Similar to infant F&Vs, the number of participants in households had an inconsistent relationship with the choice of the costliest prominent brand. In general, households with two participants or more and those with four participants were 2%–3% more likely to choose Brand A than one-participant households. However, there was no statistically significant difference between three-participant and one-participant households. The observed relationships between the choice of Brand A and both the urban/rural status of retail chains and the length of household participation were similar for both bread and infant F&Vs.

On the other hand, many of the results for bread were inconsistent with those for infant F&Vs. For instance, minorities were significantly less likely to redeem Brand A than non-Hispanic whites. The odds of choosing Brand A among non-Hispanic black and Hispanic participants were 8% and 18% less, respectively, than among non-Hispanic white participants when examining all vendors combined. The heterogeneity of brand choice persisted across vendors. For example, non-Hispanic blacks in Vendor Sigma were 8% more likely to choose Brand A over non-Hispanic whites. The discrepancies in brand choices across language groups were similar to those across race and ethnicities.

## 4. Discussion

One key finding demonstrated by our results is that the socioeconomic contexts and vendor-specific food environments in which participants live and shop matter when considering their propensity to select the costliest prominent brands among infant F&Vs and whole grain bread. This, in turn, has implications for cost containment efforts. For instance, we observed that Hispanic participants were more likely to purchase the higher-price brand in infant F&Vs than all other race/ethnicity groups. When this characteristic was held constant, Spanish-speaking participant households were even more likely to purchase the higher-price brand. Other studies found that Spanish-speaking consumers were more likely to choose Brand 1 with a higher price partially because this brand could signal an ethnicity heritage [18]. This suggests potential culturally sensitive strategies to influence participants’ brand preferences. Standard nudging techniques (e.g., distributing guides on benefit redemption that place WIC-eligible brands in order of costliness) that may be effective in shifting choices in some groups may require additional interventions in other groups to promote any change [19,20].

Furthermore, the same group of people may make different choices in different vendor-specific store environments. For example, Hispanic participants were twice as likely to choose Brand 1 with a higher price when shopping in Vendors Beta and Sigma compared to their counterparts patronizing Alpha. One hindrance in interpreting this finding is that we are unable to discern whether the effect is due to the choice architectures employed by the individual store chains or the selection bias among the participants [20,21]. For instance, Vendor Alpha may attract bargain shoppers who, in turn, are predisposed to choose the cheaper infant F&Vs brand. Thus, it may be less worthwhile pursuing nudging cost containment strategies in Vendor Alpha than in other stores, given that the probability of a participant choosing the higher-price Brand 1 is the lowest in Vendor Alpha [17]. Inner cities are widely viewed as “food deserts”, where low-cost nutritious foods are almost unavailable [22]. Therefore, WIC participants may form a shopping preference for lower-cost food. They are more likely to choose the lower-price brand even when the higher-price brands are free in their WIC food package [23]. Nevertheless, the observed variation between vendors in term of the propensity to choose costly brands requires policymakers to consider specific food environments, including the authorized vendors that are popular among a particular group of participants and the rural/urban designation of the setting, when developing any policy that influences the choice of WIC-eligible brands.

In addition, our results show that insights gleaned from studying one food category cannot be readily applied to others. For instance, while minorities were most likely to choose the more costly brand of infant F&Vs, white participants were significantly more prone to choose the costliest prominent brand of whole wheat bread. This suggests that potential cost containment interventions could be food-category-specific.

One limitation of this study is that some relevant socio-demographics and vendor characteristics that may be related to the brand choice were not available. For example, the real-time brand availability in a vendor was unknown. However, in any given month, 87.9% of the vendors were observed redeeming WIC benefits on infant F&Vs for Brand 1 or Brand 2, and 92.7% had sold Brand A or other brand bread. Therefore, it is unlikely our results are driven by whether or not the costliest prominent brand was present in a given vendor. However, as the first study in the field that uses the universe of WIC redemptions at the state level, our results still shed important light on brand choices among WIC participants in Virginia. Policy makers can leverage our results to, for example, develop customized strategies to nudge participants to redeem the least costly brands. By applying the statistical methods and findings presented herein, the Virginia WIC program could identify participant subgroups that are the appropriate targets for such interventions. For example, nudging interventions may better target the communities statistically more prone to choose costlier brands or the authorized vendors who tend to sell costlier brands to WIC participants. In addition, more ethnic-sensitive interventions could be developed, e.g., introducing or describing lower-price brands to Hispanic participants in Spanish.

Future research should ideally study participants’ reactions to store environments and layouts of authorized vendors. Two promising approaches from the marketing literature are to focus on non-price promotions and visual merchandising management (VMM) [24]. To study non-price promotion, for example, researchers could test whether or not offering free samples of cheaper brands might “reset” consumers’ default brands when redeeming WIC benefits. To test nudge redemption behaviors using VMM strategies, researchers could study the effect on participants’ brand choices of using different types of labels and signage (e.g., brighter colors) placed under lower-priced brands. VMM techniques can help direct participants’ attention to less costly brands and prevent long in-store browsing, persuading the participants to purchase less costly brands. In addition to behavioral economics approaches, researchers could test the viability of WIC clinics, which traditionally provide on-site education about nutrition and healthy eating, incorporating education on brand choices. This may lead participants to purchase less costly brands with equivalent nutritional content, which may improve their food budget management skills after they leave the WIC program. Although encouraging the lower-price food items may perpetuate the stigma of grocery shopping in low income households, it may conversely help participants manage their non-WIC food expenses as they acknowledge that store brands may have comparable quality to national brands.

## 5. Conclusions

This study represents an early effort to answer policy-relevant research questions with recently available WIC EBT data in an ethnically diverse WIC program context. In addition to emerging as a promising source of rich data for WIC researchers, such data can be leveraged by WIC state agency managers themselves to improve the effectiveness and efficiency of program operations. This work provides an example of such an application, determining which particular participant and vendor characteristics are statistically significant predictors of brand choices. Our analysis provides a first-step toolkit that can be applied in any WIC EBT state to identify the targets where interventions are likely to have the greatest cost savings impact.

## Figures and Tables

**Table 1:** Distribution of major brands (≥1% WIC redemption share) in infant fruits & vegetables and bread.

Food Category	Brands	Total Redemption $	Store Brand
Infant Fruits and Vegetables (4 oz.)	Brand 1	$1,550,215	No
	Brand 2	$643,360	No
Bread (16 oz.)	Brand A	$1,131,886	No
	Brand B	$535,164	No
	Brand C	$246,301	No
	Brand D	$204,041	No
	Brand E	$138,830	Yes
	Brand F	$41,828	No
	Brand G	$32,743	No
	Brand H	$29,779	No
	Brand I	$15,717	Yes
	Brand J	$12,145	Yes

**Table 2:** Socio-demographics, redemption statistics of WIC participants in Virginia between May 2014 and February 2015.

Summary Measure	Infant Fruits & Vegetables				Bread			
	% of Households	% of Redemption Units	Total Cost	% Redeeming Brand 1	% of Households	% of Redemption Units	Total Cost	% Redeeming Brand A
	40,285	3,153,609	$2,109,324	62.4	99,980	1,622,353	$5,764,018	40.1
# of WIC Participants							$5,764,018	
*N* = 1	10.9	7.4	$152,432	61.2 *	32.7	24.3	$1,402,209	39.7 *
*N* = 2	55.6	56.5	$1,193,028	62.4 *	43.4	38.4	$2,217,160	40.2 *
*N* = 3	27.1	29.0	$613,442	62.7 *	19.4	28.0	$1,614,943	40.0 *
*N* ≥ 4	6.4	7.1	$150,422	63.3 *	4.5	9.2	$529,706	41.6 *
Race/Ethnicity							$5,663,760	
Non-Hispanic White	32.9	35.6	$719,870	56.6 *	32.9	34.6	$1,925,795	45.4 *
Non-Hispanic Black	32.0	29.3	$610,698	67.2 *	31.0	26.2	$1,468,938	40.9 *
Hispanic	17.2	17.8	$383,667	68.2 *	21.5	24.3	$1,428,209	31.4 *
Other	15.8	17.3	$358,828	62.5 *	12.3	14.9	$840,818	37.3 *
Language							$5,764,018	
English	83.8	83.0	$1,733,522	61.6 *	80.7	76.9	$4,373,010	43.1 *
Spanish	14.4	15.1	$334,183	70.0 *	17.2	20.3	$1,228,835	30.1 *
Other	1.8	2.0	$41,620	62.6 *	2.0	2.8	$162,173	30.2 *
Participation Periods							$5,764,018	
≤3 months	9.7	5.1	$105,103	54.2 *	20.4	6.5	$367,377	40.4 *
>3–≤6 months	17.7	15.0	$313,576	54.7 *	22.8	16.9	$967,511	41.2 *
>6–≤9 months	44.9	46.6	$983,165	55.5 *	38.0	46.8	$2,694,326	40.8 *
>9 months	27.6	33.3	$707,481	58.0 *	18.8	29.7	$1,734,804	38.4 *

* *p* < 0.001.

**Table 3:** Mixed effects logistic regression results of the selection of Brand 1 in infant fruits and vegetables among WIC participants in Virginia.

Brand 1 = 1	All Stores		Vendor Alpha		Vendor Beta		Vendor Sigma	
	OR	95% CI	OR	95% CI	OR	95% CI	OR	95% CI
# of WIC-Ps								
*N* = 1	Ref		Ref		Ref		Ref	
*N* = 2	0.99	(0.98, 1.02)	0.98	(0.95, 1.01)	1.03 **	(1.00, 1.06)	0.98	(0.94, 1.03)
*N* = 3	1.01	(0.98, 1.02)	0.99	(0.96, 1.02)	1.08 ***	(1.05, 1.12)	0.87 ***	(0.83, 0.92)
*N* ≥ 4	1	(0.98, 1.02)	1.02	(0.99, 1.06)	1.07 ***	(1.03, 1.12)	0.84 ***	(0.79, 0.89)
Race								
Non-Hispanic White	Ref		Ref		Ref		Ref	
Non-Hispanic Black	1.27 ***	(1.25, 1.28)	1.20 ***	(1.19, 1.23)	1.49 ***	(1.46, 1.51)	1.18 ***	(1.14, 1.22)
Hispanic	1.14 ***	(1.12, 1.16)	1.08 ***	(1.04, 1.11)	1.25 ***	(1.21, 1.29)	1.31 ***	(1.23, 1.38)
Other	1.11 ***	(1.10, 1.12)	1.06 ***	(1.04, 1.08)	1.23 ***	(1.20, 1.25)	1.10 ***	(1.06, 1.13)
Language								
English	Ref		Ref		Ref		Ref	
Spanish	1.21 ***	(1.18, 1.23)	1.18 ***	(1.14, 1.23)	1.28 ***	(1.24, 1.33)	1.14 **	(1.05, 1.23)
Other	1.13 ***	(1.10, 1.17)	1.44 ***	(1.30, 1.60)	1.11 ***	(1.05, 1.18)	1.43 ***	(1.31, 1.57)
Participation Periods								
≤3 months	Ref		Ref		Ref		Ref	
>3–≤6 months	1.03 **	(1.01, 1.05)	0.99	(0.96, 1.03)	1.02	(0.98, 1.05)	1.07 *	(1.01, 1.14)
>6–≤9 months	1.01	(0.99, 1.03)	0.98	(0.95, 1.01)	0.99	(0.96, 1.02)	1.1 **	(1.03, 1.16)
>9 months	1.00	(0.98, 1.02)	0.98	(0.95, 1.01)	0.93 ***	(0.90, 0.96)	1.08 **	(1.02, 1.25)
Urban	0.91 ***	(0.89, 0.91)	0.98	(0.92, 1.03)	0.89 **	(0.82, 0.90)	0.85 **	(0.71, 0.97)
Store size								
Large	Ref		N/A	N/A	N/A	N/A	N/A	N/A
Mid-size	0.95 ***	(0.93, 0.97)	N/A	N/A	N/A	N/A	N/A	N/A
Small	1.37 ***	(1.24, 1.50)	N/A	N/A	N/A	N/A	N/A	N/A

* *p* < 0.05; ** *p* < 0.01; *** *p* < 0.001.

**Table 4:** Mixed effects logistic regression results of the selection of Brand A bread among WIC participants in Virginia.

Brand A = 1	All Stores		Vendor Alpha		Vendor Beta		Vendor Sigma	
	OR	95% CI	OR	95% CI	OR	95% CI	OR	95% CI
# of WIC-Ps								
*N* = 1	Ref		Ref		Ref		Ref	
*N* = 2	1.02 *	(1.00, 1.03)	1.01	(0.99, 1.03)	1.09 ***	(1.06, 1.13)	0.92 ***	(0.88, 0.96)
*N* = 3	0.99	(0.97, 1.01)	1.03 *	(1.00, 1.05)	1.01	(0.97, 1.04)	0.95 *	(0.90, 1.00)
*N* ≥ 4	1.03 **	(1.01, 1.07)	1.02	(0.98, 1.05)	1.04	(0.99, 1.09)	1.07	(0.99, 1.14)
Race								
Non-H White	Ref		Ref		Ref		Ref	
Non-H Black	0.92 ***	(0.91, 0.94)	1.02	(0.99, 1.05)	0.97	(0.94, 1.00)	1.08 **	(1.03, 1.14)
Hispanic	0.82 ***	(0.80, 0.83)	0.93 ***	(0.89, 0.96)	0.88 ***	(0.84, 0.92)	0.94	(0.87, 1.02)
Other	0.89 ***	(0.87, 0.91)	1.01	(0.97, 1.03)	0.97	(0.93, 1.02)	0.96	(0.90, 1.01)
Language								
English	Ref		Ref		Ref		Ref	
Spanish	0.81 ***	(0.78, 0.85)	0.92 ***	(0.88, 0.95)	0.93 ***	(0.88, 0.89)	1.01	(0.92, 1.12)
Other	0.87 ***	(0.85, 0.89)	1.03	(0.94, 1.13)	0.89 **	(0.81, 0.98)	1.24 ***	(1.11, 1.38)
Participation Periods								
≤3 months	Ref		Ref		Ref		Ref	
>3–≤6 months	1.02	(1.00, 1.05)	1.02	(0.98, 1.06)	1.01	(0.96, 1.07)	1.16 ***	(1.07, 1.26)
>6–≤9 months	1.04 **	(1.01, 1.06)	1.05 **	(1.01, 1.09)	0.94 *	(0.90, 1.00)	1.32 ***	(1.22, 1.42)
>9 months	0.99	(0.96, 1.01)	1.08 ***	(1.04, 1.12)	0.98	(0.93, 1.04)	1.44 ***	(1.32, 1.56)
Urban	0.91 ***	(0.90, 0.93)	0.97	(0.77, 1.13)	0.69	(0.18, 1.06)	1.19	(0.99, 1.49)
Store size								
Large	Ref		N/A		N/A		N/A	
Mid-size	0.87 ***	(0.85, 0.90)	N/A		N/A		N/A	
Small	0.96	(0.86, 1.07)	N/A		N/A		N/A	

* *p* < 0.05; ** *p* < 0.01; *** *p* < 0.001.
